# Prospective Associations Between Fixed-Term Contract Positions and Mental Illness Rates in Denmark’s General Workforce: Protocol for a Cohort Study

**DOI:** 10.2196/24392

**Published:** 2021-02-05

**Authors:** Harald Hannerz, Hermann Burr, Helle Soll-Johanning, Martin Lindhardt Nielsen, Anne Helene Garde, Mari-Ann Flyvholm

**Affiliations:** 1 The National Research Center for the Working Environment Copenhagen Denmark; 2 Bundesanstalt für Arbeitsschutz und Arbeitsmedizin Berlin Germany; 3 Lægekonsulenten dk Viby J Denmark

**Keywords:** cohort study, fixed-term employment, fixed term contract, unemployment, psychotropic drugs, psychiatric hospital treatment

## Abstract

**Background:**

In 2018, 14% of employees in the European Union had fixed-term contracts. Fixed-term contract positions are often less secure than permanent contract positions. Perceived job insecurity has been associated with increased rates of mental ill health. However, the association between fixed-term contract positions and mental ill health is uncertain. A recent review concluded that the quality of most existing studies is low and that the results of the few studies with high quality are contradictory.

**Objective:**

This study aims to estimate the incidence rate ratios (RRs) of psychotropic drug use and psychiatric hospital treatment. These ratios will be considered, first, in relation to the contrast *fixed-term versus permanent contract* and, second, to *fixed-term contract versus unemployment*.

**Methods:**

Interview data with baseline information on employment status from the Danish Labor Force Surveys in the years 2001-2013 will be linked to data from national registers. Participants will be followed up for up to 5 years after the interview. Poisson regression will be used to estimate incidence RRs for psychiatric hospital treatment for mood, anxiety, or stress-related disorders and redeemed prescriptions for psychotropic drugs, as a function of employment status at baseline. The following contrasts will be considered: full-time temporary employment versus full-time permanent employment and temporary employment (regardless of weekly working hours) versus unemployment. The analyses will be controlled for a series of possible confounders. People who have received sickness benefits, have received social security cash benefits, have redeemed a prescription for psychotropic drugs, or have received psychiatric hospital treatment for a mental disorder sometime during a 1-year period preceding baseline will be excluded from the study. The study will include approximately 134,000 participants (13,000 unemployed, 106,000 with permanent contracts, and 15,000 with fixed-term contracts). We expect to find approximately 16,400 incident cases of redeemed prescriptions of psychotropic drugs and 2150 incident cases of psychiatric hospital treatment for mood, anxiety, or stress-related disorders.

**Results:**

We expect the analyses to be completed by the end of 2021 and the results to be published in mid-2022.

**Conclusions:**

The statistical power of the study will be large enough to test the hypothesis of a prospective association between fixed-term contract positions and mental illness in the general workforce of Denmark.

**International Registered Report Identifier (IRRID):**

DERR1-10.2196/24392

## Introduction

### Background

Mental health problems are the most frequent single cause of disability benefits in the Organization for Economic Co-operation and Development (OECD) [1]. In Denmark, mental health problems account for almost half of all new applications for disability benefits [1].

The prevalence of temporary employment contracts in the European Union (EU) has been quite stable since 2005 [2]. Approximately 14% of employees (27 million persons) in the EU had a fixed-term contract in 2018 [3]. The total cost of mental health illness in the EU-28 nations was estimated to be approximately 600 billion euros in 2015, which corresponds to 4.1% combined gross domestic product of the 28 nations [4]. It has been hypothesized that some cases of mental health illness may be due to insecure employment contracts [5]. The main reason for suspecting a link between fixed-term contracts and mental health problems is that perceived job insecurity has been associated with an increased prevalence of depression, anxiety, emotional exhaustion, life satisfaction, and psychological well-being [6] as well as an increased risk of developing depressive symptoms [7]. In addition, employees in fixed-term contracts often have less influence on workplace decisions than employees with permanent contracts [8,9]. Furthermore, a low decision latitude has been associated with an increased risk of developing clinical depression [10].

However, it is still not clear whether fixed-term contracts pose a risk for poor mental health. A review by Hünefeld and Köper [5] considered 84 estimated associations between fixed-term versus permanent contract and mental health. Statistical significance was reported in 40% of the included studies, but only half of the significant associations were positive. Moreover, a recent systematic review and meta-analysis of studies on fixed-term versus permanent contracts and mental health problems concluded (1) that the quality of most existing studies was low and (2) that the results of the few studies with sufficient quality were contradictory [11].

The association between unemployment and mental health disorders has been robustly researched and published. There is a consensus that people who are unemployed are at increased risk of developing mental health problems and that employees with mental health problems are at increased risk of becoming unemployed [12]. A substantial reason for the association between unemployment and mental illness is attributed to the mental distress of chronic financial insecurity [7]. From this perspective, it has been hypothesized that the anticipation of a job loss can be detrimental to mental health as unemployment itself [7]. Recently, a meta-analysis was carried out to estimate the relative risk of developing depression as a function of unemployment and self-perceived job insecurity [7]. The study included results from 20 cohort studies, of which 14 compared the risk among unemployed with the risk among employees, whereas 6 compared the risk among employees with perceived job insecurity with that among other employees. The odds ratio for the contrast *unemployment versus employment* was estimated to be 1.19 (95% CI 1.11‑1.28), whereas the odds ratio for “secure versus unsecure employment” was estimated to be 1.29 (95% CI 1.06‑1.57).

### Objectives

This project aims to estimate the incidence rate ratios (RRs) of psychotropic drug usage and psychiatric hospital treatment. These ratios will be considered in relation to the contrast “fixed-term versus permanent contract” and to “fixed-term contract (regardless of weekly working hours) versus unemployment” among the general population of Denmark. The second analysis will be performed to elucidate the hypothesis of Kim and von dem Knesebeck [7], which states that the anticipation of a possible job loss can be as detrimental for the mental health as unemployment itself.

People may work part time due to health issues or because they are not able to find a full-time job. They may also have chosen to work part time, for example, for furthering their education, caring for a parent or child, or engaging in hobbies or sports activities. The reason for excluding part-time workers in the first analysis is that a participant may have chosen to work part time due to ill health. The reason for not excluding part-time workers in the second analysis is that our data do not permit differentiation between part-time and full-time unemployment.

Job insecurity and unemployment have been associated with an increased risk of mental distress from chronic financial insecurity, which in turn has been associated with an increased risk of mental illness [13-15]. It is reasonable to believe that most people are financially more secure in a fixed-term contract position than they are in a state of unemployment. From this viewpoint, we expect the risk of developing mental health illnesses to be higher among unemployed people than among employees with a fixed-term contract. Likewise, we expect the risk to be higher among employees with a fixed-term contract than among employees with a permanent contract.

## Methods

### Ethics Approval

The study will comply with The Act on Processing of Personal Data, Denmark (Act No. 429 of May 31, 2000), which implements the European Union Directive 95/46/EC on the protection of individuals. The data usage was approved by the Danish Data Protection Agency (file number 2001-54-0180). The ethical and legal aspects of the project were approved by Statistics Denmark, accounting for 704291. In Denmark, register studies, which do not include medical procedures, are not part of the ethical committee system.

### Data Sources

All residents of Denmark have access to tax-financed health care. The educational system is generally tax financed. The so-called flexicurity model provides an income safety net for the unemployed, with unemployment insurance benefits for members of unemployment insurance funds. The residents of Denmark are also entitled to maternity and paternity benefits, sickness-absence benefits, disability benefits, and if needed, social security cash benefits. Person-based data on health care services and redeemed prescriptions of medicine and welfare benefits payments are collected and reported in national registers, with unique personal identification numbers, which are assigned to all residents of Denmark [16].

This study will be based on baseline data on employment status from the Danish Labor Force Survey (DLFS) 2001-2013 and follow-up data on health from a series of registers, which cover the entire population of Denmark. The following registers will be used: the Central Person Register (CPR) [17], the Employment Classification Module (ECM) [18], the Danish Education Registers [19], the Danish Family Income Register [20], the Danish Register for Evaluation of Marginalization (DREAM) [21], the Psychiatric Central Research Register [22], and the National Prescription Register [23]. Linkage will be based on participants’ personal identification numbers. The data sources and information to be included are listed in Table 1.

**Table 1:** The data sources of the project.

Data source	Type of data source	Information to be included in the present project
The Danish Labor Force Survey [24]	Survey data obtained from interviews on random samples of the population of Denmark	Date of the interview, employment status, type of employment contract, and nighttime work
The Central Person Register [17]	National register, which covers all residents of Denmark	Gender, age, date of migration, and date of death
The Employment Classification Module [18]	National register, which covers all residents of Denmark	Industry sector
The Danish Education Registers [19]	National register, which covers all residents of Denmark	Educational level
The Danish Family Income Register [20]	National register, which covers all residents of Denmark	Equalized disposable family income
The Danish Register for Evaluation of Marginalisation [21]	National register, which covers all residents of Denmark	Date of welfare benefits payment and type of welfare benefits payment
The Psychiatric Central Research Register [22]	National register, which covers all residents of Denmark	Date of hospital contact and principal diagnosis (ICD-10^a^ code)
The National Prescription Register [23]	National register, which covers all residents of Denmark	Date of redeemed prescription and type of medicine (ATC^b^-code)

^a^ICD-10: International Statistical Classification of Diseases and Related Health Problems, 10th Revision.

^b^ATC: Anatomical Therapeutic Chemical Classification System.

DLFS is based on quarterly random samples of 15- to 74-year-old residents of Denmark, with systematic oversampling of unemployed people. Each participant is invited to be interviewed 4 times over the course of a year and a half. The purpose of the interviews is to collect person-based information on inter alia, labor market attachment, type of contract, and working hours [24]. Among those invited for the DFLS, the response rate decreased over time from 70% in 2002 to 53% in 2013 [25]. The CPR contains, inter alia, information on gender, addresses, and dates of birth, death, and migrations for every person who is or has been a resident of Denmark sometime between 1968 and the present time. The ECM contains annual, person-based information on, inter alia, the socio-economic status, occupation, and industry of the residents of Denmark. The Danish Education Registers contain person-based information on, inter alia, a person’s highest educational attainment. The Danish Family Income Register contains information on household income. DREAM contains weekly, person-based information on social transfer payments (welfare benefits payments) such as maternity and paternity benefits, sickness-absence benefits, unemployment benefits, social security cash benefits, and state educational grants. DREAM has existed since 1991 and covers all residents of Denmark. The weekly benefits data are recorded if the person has been on a benefit for 1 or more days of the week. However, as only 1 type of social transfer payment can be registered per week, types of benefits are prioritized in the case of data overlap. The above-mentioned social transfer payments are prioritized in the order listed, that is, maternity and paternity benefits have higher priority than sickness-absence benefits, which in turn have higher priority than unemployment benefits, etc. The Psychiatric Central Research Register contains person-based information on inpatients, outpatients, and emergency ward visits in all psychiatric hospital departments in Denmark. The National Prescription Register contains person-based data on all redeemed prescriptions at pharmacies in Denmark.

This study has access to the data on Anatomical Therapeutic Chemical Classification System (ATC) codes [26] from the National Prescription Register for the time period 2000-2014 and International Statistical Classification of Diseases and Related Health Problems, 10th Revision (ICD-10) codes [27] from the Psychiatric Central Research Register for the time period 1995-2017.

#### Clinical Endpoints

RRs will be examined for the following endpoints:

- Redeemed prescriptions for any type of psychotropic medicine, that is, drugs in the ATC-code category N05 (psycholeptica) or N06 (psychoanaleptica)
- Psychiatric hospital treatment with mood, anxiety, or stress-related disorder (ICD-10: F30–F41 or F43) as the principal diagnosis

The following mental disorders are included in the above case definition:

- F30 Manic episode
- F31 Bipolar affective disorder
- F32 Depressive episode
- F33 Recurrent depressive disorder
- F34 Persistent mood (affective) disorders
- F38 Other mood (affective) disorders
- F39 Unspecified mood (affective) disorder
- F40 Phobic anxiety disorders
- F41 Other anxiety disorders
- F43 Reaction to severe stress and adjustment disorders

#### Exposure

The following exposure categories will be considered: *unemployed but actively searching for a job and ready to start working within 14 days*, *employed on a fixed-term contract position*, and *employed on a permanent contract*. The categories are based on the DLFS-questionnaire [18].

### Covariates

The literature suggests that estimated risks of mental health depend on gender [28,29], age [30-32], calendar year [33], education level [34], and income [35-38]. Moreover, it has been shown that the birth of a child may result in maternal [39] and paternal [40] postpartum depression.

The following covariates will therefore be regarded in all analyses: gender, age, calendar time of the interview, education level (at the end of the calendar year preceding the interview), equivalent disposable family income (in the calendar year preceding the interview), and maternity or paternity benefits (in the 1-year period preceding the interview).

In addition to the above, the following covariates will be regarded in the analyses of differences between employees with fixed-term and permanent contracts: main industry (in the calendar year preceding the interview), unemployment benefits (in the 1-year period preceding the interview), state educational grants (in the 1-year period preceding the interview), and nighttime work (at the time of the interview). We control for industry, as a previous study has found significant industry-related inequalities in the rate of mood disorders among employees in the general working population of Denmark [41]. We control for unemployment benefits and state educational grants in the 1-year period preceding the interview, as we believe that people’s attitudes toward fixed-term and permanent contracts may depend on their previous labor market attachment. We control for nighttime work because it has been shown that the prevalence of psychotropic drug usage in Denmark is greater among shift workers than among workers without shift work [42].

The variables will be operationalized as follows:

#### Gender

Gender is classified into male or female as registered in the CPR.

#### Age

In this study, we will not have access to the exact dates of birth, but we will have access to information about the birth year of the participants, that is, we will know what their integer age was at the very beginning and at the very end of a calendar year. To form baseline age categories, the participants who were interviewed before July 1 in a given calendar year will be assigned the integer age they had at the beginning of that year, whereas the participants who were interviewed after June 30 will be assigned the integer age they would have at the end of the calendar year. The participants will thereafter be divided into 10-year age groups (20-29,…50-59 years), and the age group will be treated as a categorical variable.

#### Calendar Time of the Interview

The calendar years of the interviews will be treated as a categorical variable and divided into the following categories: 2001-2003, 2004-2006, 2007-2009, and 2010-2013.

#### Educational Level (at the End of the Calendar Year Preceding the Interview)

A person’s highest attained education is registered and classified with a 2-digit code in the Danish Education Registers [19]. In this study, it will be divided as follows (Table 2) into the categories low, medium, high, and unstated.

**Table 2:** Classification of education levels.

The present project	The Danish Education Registers
Low	10 Primary and lower secondary education
Medium	20 Upper secondary education 30 Basic vocational education 35 Qualifying vocational education 40 Short-term tertiary education
High	50 Medium-term tertiary education 60 Bachelor’s degree 70 Master’s degree or equivalent tertiary education level 80 Doctoral degree or equivalent tertiary education level
Unstated	Unstated

#### Equivalized Disposable Family Income (in the Calendar Year Preceding the Interview)

The equivalent disposable income is the total income of a household, after tax and other deductions, which is available for spending or saving, divided by the number of household members converted into equalized adults; household members are equalized or made equivalent by weighting each according to their age, using the so-called modified OECD equivalence scale (cited from Eurostat [43]).

The equivalent disposable income is calculated in 3 steps (cited from Eurostat [43]):

- All monetary incomes received from any source by each member of a household are added up. These include income from work, investment, and social benefits, as well as any other household income; taxes and social contributions that have been paid are deducted from this sum.
- To reflect differences in household size and composition, the total (net) household income is divided by the number of ‘equivalent adults,’ using a standard (equivalence) scale: the modified OECD scale. This scale gives a weight to all members of the household (and then adds these up to arrive at the equivalized household size): 1.0 to the first adult, 0.5 to the second and each subsequent person aged 14 and over, and 0.3 to each child aged under 14.
- Finally, the resulting figure is called the equivalent disposable income and is attributed equally to each member of the household.

This study will treat the equivalent disposable family income as a categorical variable, divided into low, medium, and high in accordance with calendar-year specific sample tertiles. The tertiles will be based on all DLFS responders who were 20 to 59 years old and employed at the time of the interview.

#### Main Industry (in the Calendar Year Preceding the Interview)

The industries will be divided into 10 groups, as shown in Table 3. The industrial codes are based on the industrial classification DB93 [44] in 1999-2002, DB03 [45] in 2002-2007, and DB07 [46] in the calendar years 2008-2013.

**Table 3:** Industrial groups coded according to the classifications DB93, DB03, and DB07, respectively.

Industrial group	Code according to		
	DB93	DB03	DB07
Agriculture, forestry, hunting, and fishing	A+B	A+B	A
Manufacturing, mining, and quarrying	C+D	C+D	B+C
Construction	F	F	F
Wholesale and retail trade and repair of motor vehicles and motorcycles	G	G	G
Transporting and storage	I	I	H
Accommodation and food service activities	H	H	I
Human health and social work activities	N	N	Q
Other	E, J, K, L, M, O, P, Q	E, J, K, L, M, O, P, Q	D, E, J, K, L, M, N, O, P, R, S, T, U
Unstated	X	Missing	Missing

#### Unemployment Benefits (in the One-Year Period Preceding the Interview)

This variable is equal to 1 if the participant, according to DREAM, received unemployment benefits (DREAM codes: 111-115, 121-126, 211-219, 231, 232, and 299) at least once during the 1-year period preceding the DLFS interview. Otherwise, it is equal to 0.

#### Maternity or Paternity Benefits (in the One-Yar Period Preceding the Interview)

This variable is equal to 1 if the participant, according to DREAM, received maternity or paternity benefits (DREAM code: 881) at least once during the 1-year period preceding the DLFS interview. Otherwise, it is equal to 0.

#### State Educational Grants (in the One-Year Period Preceding the Interview)

This variable is equal to 1 if the participant, according to DREAM, received state educational grant payments (DREAM codes: 651, 652, and 661) at least once during the 1-year period preceding the DLFS interview. Otherwise, it is equal to 0.

#### Nighttime Work

In the DLFS interview, the participants were asked whether they worked at night during the last 4 weeks. In this study, nighttime work will be treated as a categorical variable in accordance with the 3 response categories *Yes, regularly*, *Yes, occasionally*, and *No*.

### Follow-Up

The study will be based on data that already exist. The included participants of the DLFS will be followed by national registers. The follow-up in the register data will start on the date when 6 weeks would have passed since the first DLFS interview and end on the date when any of the following events occur: 5 years pass since the date of the start of the follow-up, the participant emigrates, the participant dies, the participant meets the clinical endpoint of the analysis, or the study period ends. The end of the study period was set at the end of the calendar years 2014 and 2017 for redeemed prescriptions of psychotropic drugs and psychiatric hospital treatments, respectively. Person-years at risk will be calculated for each of the included participants. Participants who die or emigrate during the follow-up will be censored at the time of the event. That is, they will participate with person-years at risk until the time of death or emigration.

### Inclusion Criteria

The primary analyses will be based on data from the participants’ first interview in the time period 2001‑2013. In the comparisons between employees with a full-time fixed-term versus a full-time permanent contract, we will require inclusion criteria 1-6 fulfilled (see below). In the comparisons between employees with a fixed-term contract (regardless of weekly working hours) versus unemployed people, we will require that criteria 1-4 and criteria 7 are fulfilled.

Inclusion criteria:

1. The participants were aged between 20 and 59 years at the time of the interview.
2. They did not receive any social transfer payments other than holiday allowance (DREAM code: 121), unemployment benefits (DREAM codes: 111-115, 122-126, 211-219, 231, 232, 299), maternity or paternity benefits (DREAM code: 881), or state educational grants (DREAM codes: 651, 652, 661) during the one-year period preceding the interview.
3. They did not receive any psychiatric hospital treatment with mental disorders (ICD-10: F01–F99) as the principal diagnosis during a 1-year period preceding the start of follow-up.
4. They did not redeem any prescription for psychotropic drugs (ATC: N05–N06) during a 1-year period preceding the start of follow-up.
5. They were employees, according to the interview.
6. They usually worked ≥32 h a week, according to the interview.
7. They were either unemployed but actively searching for a job and ready to start working within 14 days or employed with a fixed-term contract at the time of the interview.

Moreover, it is necessary that the concerned DLFS–based exposure variables and covariates are nonmissing.

### Primary Statistical Analysis

Poisson regression will be used to estimate incidence RRs for psychiatric hospital treatment for mood, anxiety, or stress-related disorders and redeemed prescriptions for psychotropic drugs, as a function of employment status at baseline. The following contrasts will be considered: (1) Full-time fixed-term contract versus full-time permanent contract and (2) fixed-term contract (regardless of weekly working hours) versus unemployment. All analyses will be controlled for age, sex, disposable family income, educational level, calendar year of the interview, and reception of maternity or paternity benefits sometime during a 1-year period preceding baseline. The RRs for the contrast *fixed-term versus permanent contract* will, in addition to the above, be controlled for baseline industry group and nighttime work as well as reception of unemployment benefits and state study grants, sometime during a 1-year period preceding baseline. The logarithm of person-years at risk will be used as an offset. Likelihood ratio tests will be used to test first for main effects and then for effects of interaction with gender, age, and education level. We test for interactions, as it has been suggested that the strength of adverse health effects of fixed-term contracts depends on gender [47], age [48], and education level [49].

The main effects will be tested both for psychiatric hospital treatments and redeemed prescriptions for psychotropic drugs. The interaction effects will only be tested for redeemed prescriptions for psychotropic drugs. A Bonferroni correction will be used to adjust for multiple testing. We want the overall significance level to be less than or equal to 0.05. Hence, each of the 10 tests will be conducted at a significance level of 0.005. RRs for main effects will be estimated and presented with 99.5% CI. Moreover, the RRs for redeemed prescriptions for psychotropic drugs will be stratified (and presented with 99.5% CI) by gender, age, and educational level.

### Power Calculations

Under the null hypothesis, we expected to find approximately 29 new cases of psychotropic drug usage and 3.4 new cases of psychiatric hospital treatment for mood, anxiety, or stress-related disorders per 1000 person-years at risk [50]. If we assume that approximately 15% of the otherwise eligible participants will be excluded due to exclusion criteria 1-4, the total number of expected cases in the concerned exposure categories will be approximately as shown in Table 4.

**Table 4:** The total number of expected cases in the concerned exposure categories.

Exposure category	Expected number of eligible participants	Expected number of psychotropic drug cases	Expected number of psychiatric hospital cases
Fixed-term full-time contract	10,600	1300	170
Permanent full-time contract	106,000	13,000	1700
Fixed-term contract (regardless of weekly working hours)	15,000	1800	240
Unemployment	13,000	1600	210

**Figure 1:**
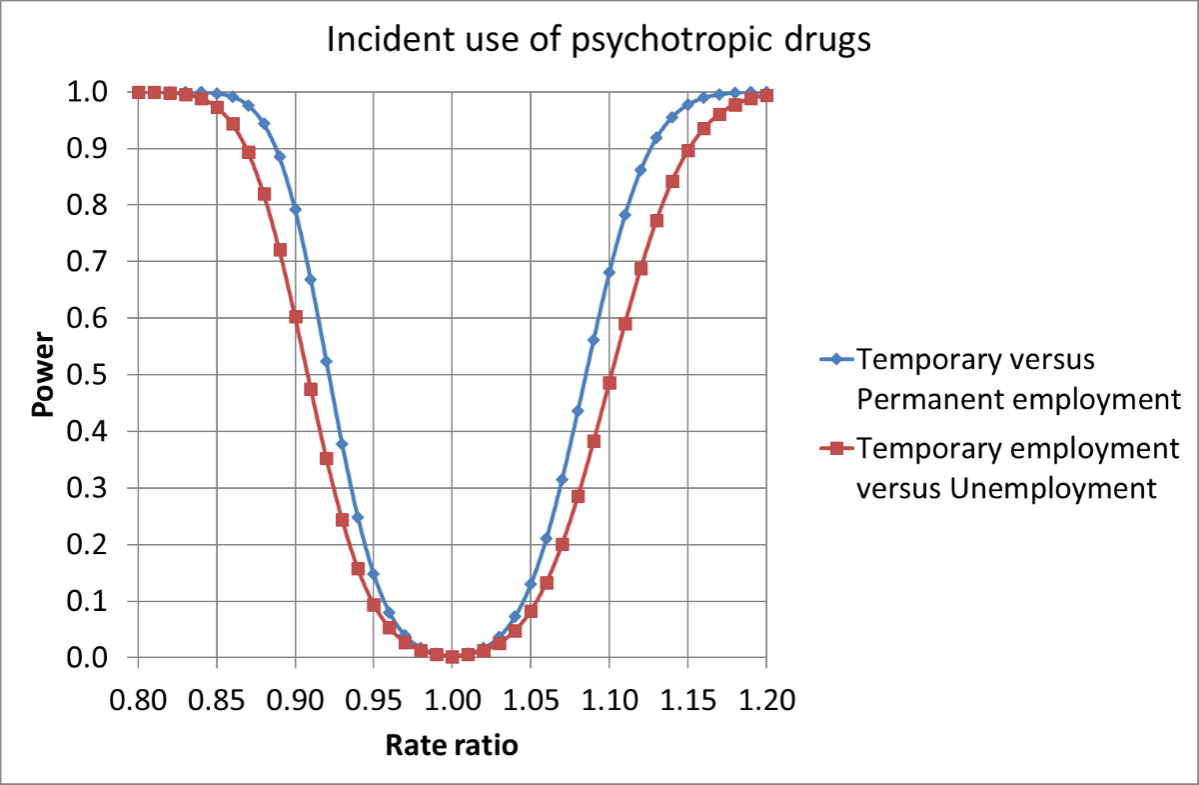
Power to detect main effects of fixed-term contracts on the rates of new cases of psychotropic drug use, as a function of underlying rate ratios (α=.005).

**Figure 2:**
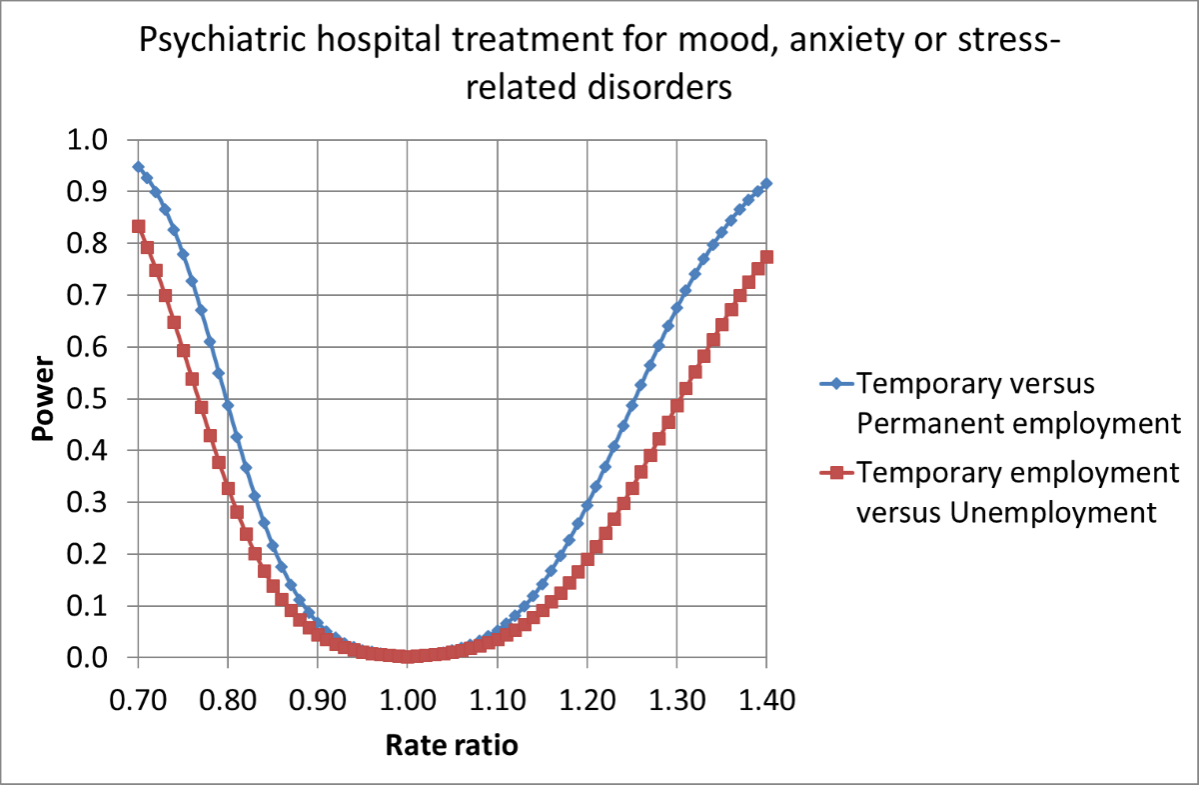
Power to detect main effects of fixed-term contracts on the rates of new cases of psychiatric hospital treatment for mood, anxiety, or stress-related disorders, as a function of underlying rate ratios (α=.005).

On the basis of the expected number of cases, the Poisson distribution, the Gauss propagation of error formulas, and the central limit theorem, we estimated the statistical power of the planned significance tests. The statistical powers for the main effects are given in Figures 1 and 2, for incident use of psychotropic drugs and psychiatric hospital treatment for mood, anxiety, or stress-related disorders, respectively, as a function of the underlying RR.

The statistical powers to detect interaction effects were estimated in relation to the Cohen w [51], where w=0.1 is defined as a small effect, w=0.3 is defined as a medium effect, and w=0.5 is defined as a large effect. On the basis of the expected number of cases, test specific degrees of freedom, and the noncentral chi-square distribution, we estimated that the power to detect a small effect (w=0.1) is greater than 0.98 in each of the planned interaction tests of this project.

The calculations indicate that the power is sufficiently large to test both the main and interaction effects of fixed-term contracts on the incidence of psychotropic drug usage. The powers to detect effects of fixed-term contracts on the incidence of psychiatric hospital treatment for mood, anxiety, or stress-related disorders (Figure 2) are, however, quite low, and this needs to be taken into account when the results are evaluated.

### Sensitivity Analyses

We will conduct 6 sensitivity analyses. The endpoint of the sensitivity analyses will be redeemed prescriptions of psychotropic drugs. Interaction effects will be disregarded. RRs will be estimated and presented with 99.5% CI. The RRs and their associated confidence intervals will not be regarded as statistical significance tests. However, they may strengthen, weaken, or invalidate the statistical conclusions of the primary analyses.

#### Sensitivity Analysis 1: Exclusion of All Cases that Occurred Within 5 Years Preceding the Start of Follow-Up

The primary analysis will exclude all people who received psychiatric hospital treatment or redeemed a prescription for psychotropic drugs sometime during a 1-year period before the start of the follow-up. Hence, no known current cases of psychiatric treatment will be included in the follow-up. It is, however, possible that people who received treatment more than 1 year before the follow-up will influence the analysis. To shed some light on this issue, we will conduct a sensitivity analysis in which we will exclude all people who received psychiatric hospital treatment or redeemed a prescription for psychotropic drugs sometime during a 5-year period before the start of the follow-up. This sensitivity analysis will be based on data from the participants’ first interview in the period 2005-2013. Moreover, it will only include people who lived in Denmark throughout the concerned 5-year period. The statistical models and inclusion criteria will otherwise be the same as in the primary analysis. The interpretation of results will include the fact that approximately 20% of the population experiences mental health problems during their lifespan due to different causes; so the analysis may be overcontrolling.

#### Sensitivity Analysis 2: Relapse Rate Ratios

At this point, it is useful to further elucidate (/examine) the possible influence of former cases of psychiatric treatment on the association between fixed-term contract and psychotropic drug usage. To this end, we will estimate relapse RRs among the participants who were excluded from the first sensitivity analysis due to psychiatric hospital treatment or redeemed prescription for psychotropic drugs sometime between 1 and 5 years before the start of follow-up.

Current cases, that is, people who received treatment sometime during a 1-year period before the start of follow-up will still be excluded. The statistical models will otherwise be the same as in sensitivity analysis 1.

#### Sensitivity Analysis 3: Long-term Exposure Versus Exposure at a Single Time Point

In the primary analysis, we regard the contrasts *full-time fixed-term contract versus full-time permanent contract* and *fixed-term contract versus unemployment* with the exposure categories defined at a single time point (the first interview). We want to know whether the strength of the concerned associations will increase if we base the exposure categories on more than one interview and only include people who belong to the same exposure category in all of their interview rounds. In other words, we want to know whether the strength of the associations will increase if we base the contrasts on *long-term exposure* instead of *exposure at a single time point*. To shed some light on this issue, we will conduct a sensitivity analysis in which we will only include people who (1) participated in more than one interview, (2) were aged between 20 and 59 years during their last interview, and (3) belonged to the same exposure category in all of their interview rounds. The follow-up of the included participants will commence 6 weeks after their last interview. The statistical models and inclusion criteria will otherwise be the same as in the primary analysis.

#### Sensitivity Analysis 4: Minimally Adjusted Rate Ratios

In the primary analyses, we will exclude all people who received sickness benefits or social security cash benefits during a 1-year period before the baseline interview. Moreover, we control for disposable family income as well as a series of other covariates. It is possible that the rigorous inclusion criteria and the many control variables will lead to overly conservative estimates. Therefore, we will conduct a sensitivity analysis in which we will (1) remove the second of the inclusion criteria listed in the method section and (2) remove all control variables except for gender, age, and education. The methods will otherwise be the same as in the primary analyses.

#### Sensitivity Analysis 5: Reason for Being on a Fixed-Term Contract

All EU-Labor Force Survey participants with a fixed-term contract are asked for the reason of having a fixed-term contract. Their answers are categorized as follows:

- It is a contract covering a period of training (apprentices, trainees, research assistants, etc)
- Person could not find a permanent job
- Person did not want a permanent job
- It is a contract for a probationary period

We want to know whether the risk of developing mental health illnesses among employees with a fixed-term contract depends on the reason for being on a fixed-term contract. To answer this question, we will estimate incidence RRs for redeemed prescriptions of psychotropic drugs as a function of the reason for being on a fixed-term contract.

Participants who did not want a permanent job (category 3) will serve as the reference group. We will include all employees on fixed-term contracts, who fulfilled inclusion criteria 1-5, as listed in the method section. The analyses will initially be conducted only with full-time employees (≥32 h a week) and then with all employees regardless of weekly working hours. The analyses will be controlled for all variables given in the *Covariate* section. The statistical model and follow-up periods will be the same as in the primary analysis.

#### Sensitivity Analysis 6: Stratification by Industry Sector

We know that the prevalence of fixed-term contracts in the Nordic countries depends on the industry sector [52], and that the rates of mood disorders in the general working population of Denmark depend on the industry sector [41]. It is possible that the effect of fixed-term contract positions on mental health illnesses also depends on the industry. Therefore, we conduct a sensitivity analysis in which we will stratify the results of the comparison between employees with a fixed-term and a permanent contract by the industry sector. The industries are grouped as shown in Table 3. The inclusion criteria and covariates will be the same as in the primary analysis.

Reasons why the association between fixed-term contracts and mental health illnesses might depend on industry could be, first, that chances for reemployment may depend on the industry sector and, second, that expectations regarding a fixed-term versus permanent contract may depend on the industry.

Another reason for stratifying by the industry sector is that the social partners might be interested in seeing the association between fixed-term contracts and mental health illnesses in their own industry sector.

#### Possible Confounding From Smoking, Being Overweight, and Obesity: a Feasibility Study

Studies have suggested that smoking habits [53,54] and being overweight [55] predict depression. It has been estimated that the risk ratio of new-onset depression is 1.46 (95% CI 1.03‑2.07) for smokers versus nonsmokers [53], 1.08 (95% CI 1.02‑1.14) for overweight versus normal weight, and 1.57 (95% CI 1.23‑2.01) for obesity versus normal weight [55]. The DLFS does not contain any information about smoking habits and body weights of the participants. Therefore, we cannot control for these factors in the analyses. However, we have access to some collateral data, which we have used to estimate to what extent and in what direction the RRs of the present project are likely to be influenced by differences in distributions of body mass index and smoking habits. The collateral data were gathered from a survey on work and health in a random sample of the Danish population in 2005. The response rate of the survey was 62% [56]. In this study protocol, we have used the survey data to estimate the prevalence of smoking, being overweight, and obesity among 20-59-year-old people in Denmark, stratified by the exposure categories of interest to this study. The crude prevalence is given in Table 5, whereas prevalence that is standardized for age, gender, and education is given in Table 6. The total sample of 20- to 59-year-old people was used as the standard population.

**Table 5:** Crude percentages of current smokers, people with moderate overweight (25≤BMI<30), and people with obesity (BMI≥30), by exposure category, in a random sample of 20- to 59-year-old people in Denmark, 2005.

Exposure category	Current smoker, n (%)	25≤BMI<30, n (%)	BMI≥30, n (%)
Fixed-term full-time contract (n=748)	221 (29.5)	231 (30.9)	72 (9.6)
Permanent full-time contract (n=8016)	2438 (30.4)	2859 (35.7)	834 (10.4)
Fixed-term contract (regardless of weekly working hours; n=908)	281 (30.9)	267 (29.4)	77 (8.5)
Unemployment (n=393)	141 (35.9)	123 (31.3)	54 (13.7)

**Table 6:** Age (10-year classes), gender and education (low, medium, and high) standardized percentages of current smokers, people with moderate overweight (25≤BMI<30), and people with obesity (BMI≥30), by exposure category, in a random sample of 20- to 59-year-old people in Denmark, 2005.

Exposure category	Current smoker, % (95% CI)	25≤BMI<30, % (95% CI)	BMI≥30, % (95% CI)
Fixed-term full-time contract	31.8 (28.4-35.6)	33.0 (29.6-36.8)	10.6 (8.5-13.3)
Permanent full-time contract	30.4 (29.4-31.4)	34.3 (33.3-35.3)	10.1 (9.5-10.8)
Fixed-term contract (regardless of weekly working hours)	33.3 (30.1-36.8)	32.5 (29.4-35.9)	9.5 (7.6-11.9)
Unemployment	36.5 (32.1-41.4)	31.9 (27.4-37.1)	14.1 (11.0-18.2)

Table 6 suggests that the standardized prevalence among people with fixed-term full-time contracts are very similar to those among people with permanent full-time contracts. We note, however, that the estimated prevalence of smoking and obesity is greater among the unemployed than among the employees on fixed-term contracts. We want a rough estimate of the effect that such differences may have on the RR of mental health illnesses among employees on fixed-term contracts versus unemployed in our target population. Therefore, we have estimated the expected RR between these exposure categories under the assumption that the groups are equal in all respects other than smoking and BMI distribution and that an RR for depression can be used as a proxy for the RR of mental health illnesses. We used the following equation:



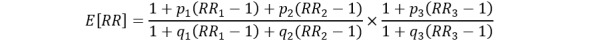



where RR_1_=1.08 is the estimated rate ratio for depression among people in the category 25≤BMI<30 versus BMI<25, RR_2_=1.57 is the estimated rate ratio for depression among people in the category BMI≥30 versus BMI<25, and RR_3_=1.46 is the estimated rate ratio for depression among smokers versus nonsmokers. The parameters p_1_, p_2_, and p_3_ are the standardized sample prevalences of overweight, obesity, and smoking, respectively, among people with fixed-term contracts (cf. Table 6). The parameters q_1_, q_2_, and q_3_ are the corresponding prevalence among the unemployed people (cf. Table 6).

The calculation yielded an estimated rate ratio of 0.96. This means that a failure to control for overweight, obesity, and smoking in this project is expected to bias the estimated rate ratio for mental health illnesses among fixed-term versus unemployed people downward with a factor of 0.96.

## Results

We expect the analyses to be completed by the end of 2021 and the results to be published mid-2022.

## Discussion

This study protocol contains a statistical analysis plan for a research project aimed at estimating prospective associations between fixed-term contracts and mental health illness in the general population of Denmark. As all covariates, outcome variables, inclusion criteria, statistical models, and significance levels are completely defined, published, and peer-reviewed before we link the exposure data of the project to its outcome data, we minimized the risk of hindsight bias.

A major strength of the project is that the data material is large enough to afford sufficient statistical power to detect important associations between fixed-term contracts and incident use of psychotropic medication. Another strength is that the outcome variables as well as the censoring variables (death and emigration) will be ascertained through national registers, which cover the entire population of Denmark. The study is weakened by the low response rate in the DLFS, which makes the representativeness of the participants questionable. Another weakness is the lack of data on lifestyle factors.

Studies have shown that the prevalence of depression tends to be higher among migrants than in the general population [57]. Studies of the general working population in Sweden [58] and Spain [59] have shown that immigrants are more likely to be on a fixed-term work contract. A survey of the general workforce in Canada found that newcomer immigrants (within the first 5 years) were on fixed-term contracts more often than natives [60]. Due to the comparability with Sweden and Canada, we expect the figures to be similar in Denmark. The response rate in questionnaire surveys among ethnic minorities in general is relatively low [61,62]; for example, half among non-Western immigrants compared with Danes [53]. The background to this study is the DLFS; thus, the issue of ethnicity cannot be addressed, as there are relatively few with immigrant background answering the survey.

Finally, it should be noted that the results of the study may not be fully transferable to other countries. Due to the relatively low employment protection in Denmark combined with a comprehensive income safety net for the unemployed, the so-called flexicurity model, it can be hypothesized that this may result in fewer fixed-term contracts in Denmark compared with other European countries [63].

## Abbreviations

ATC: Anatomical Therapeutic Chemical Classification System
CPR: Central Person Register
DLFS: Danish Labor Force Survey
DREAM: Danish Register for Evaluation of Marginalization
ECM: Employment Classification Module
EU: European Union
ICD-10: International Statistical Classification of Diseases and Related Health Problems, 10th Revision
OECD: Organization for Economic Co-operation and Development
RR: rate ratio
